# A simple and fast *Agrobacterium*-mediated transformation system for passion fruit KPF4 (*Passiflora edulis* f. *edulis* × *Passiflora edulis* f. *flavicarpa*)

**DOI:** 10.1186/s13007-020-00684-4

**Published:** 2020-10-16

**Authors:** Lydia K. Asande, Richard O. Omwoyo, Richard O. Oduor, Evans N. Nyaboga

**Affiliations:** 1 Department of Plant Science, Kenyatta University, Nairobi, P.O. Box 43844 – 00100, Kenya; 2 Department of Biochemistry, University of Nairobi, Nairobi, P.O. Box 30197 – 00100, Kenya; 3 Department of Biochemistry and Biotechnology, Kenyatta University, Nairobi, P.O. Box 43844 – 00100, Kenya

**Keywords:** *Passiflora edulis* Sims, *Agrobacterium*-mediated transformation, Transient *gus*A expression, Transformation efficiency

## Abstract

**Background:**

Passion fruit (*Passiflora edulis* Sims) is an important horticultural crop in the tropics and subtropics, where it has great commercial potential due to high demand for fresh edible fruits and processed juice as well as source of raw materials in cosmetic industries. Genetic engineering shows great potential in passion fruit improvement and can compensate for the limitations of conventional breeding. Despite the success achieved in genetic modification of few passion fruit varieties, transgenic passion fruit production is still difficult for farmer-preferred cultivars. Therefore, it is important to establish a simple and fast *Agrobacterium*-mediated cell transformation of commercial hybrid passion fruit KPF4 (*Passiflora edulis* f. *edulis* × *Passiflora edulis* f. *flavicarpa*).

**Results:**

In the present study, we have developed a simple and fast *Agrobacterium*-mediated transformation system for hybrid passion fruit KPF4 using leaf disc explants. Factors affecting the rate of transient beta (β)-glucuronidase (*gus*A) expression and consequently transformation efficiency were optimized as follows: *Agrobacterium* cell density with an OD_600_ of 0.5, 30 min infection time, 3 days of co-cultivation duration and the incorporation of 200 µM acetosyringone into *Agrobacterium* infection suspension medium. Using the optimized conditions, transgenic plants of KPF4 were produced within 2 months with an average transformation efficiency of 0.67%. The β-glucuronidase (GUS) histochemical staining confirmed the expression and integration of an intron-containing *gus*A gene into transformed leaf discs and transgenic plant lines of KPF4. The presence of *gus*A gene in the transgenic plants was confirmed by polymerase chain reaction (PCR). The results confirmed that the *gus*A gene was efficiently integrated into the passion fruit genome.

**Conclusions:**

The developed transformation protocol is simple and rapid and could be useful for functional genomic studies and transferring agronomically important traits into passion fruit hybrid KPF4. This study developed a method that can be used to transfer traits such as resistance to viral diseases, low fruit quality and short storage life. To the best of our knowledge, this is the first report on genetic transformation system for commercial passion fruit hybrid KPF4.

## Background

Passion fruit (*Passiflora edulis* Sims), a dicotyledonous perennial plant with shallow roots and woody vines, is widely cultivated in the sub-tropics and tropics [1]. It is an important fruit crop due to its nutritional, medicinal (as sedatives, antiplasmodic, and antibacterial) and ornamental value. Nutritionally, the ripe fruits are an important source of minerals and vitamins (including ascorbic acid), phytoconstituents and phenolic compounds [2, 3]. The use of passion fruit in cosmetic industry has also contributed to the increased production acreage in sub-tropical and tropical countries [4]. However, the low genetic variability, self and inter-specific incompatibility, high levels of ploidy, lack of pathogen resistance genes in the available cultivars and elimination of deleterious genes by many cycles of selfing and backcrossing hinder passion fruit improvement efforts by conventional breeding [5, 6]. To circumvent the limitations of conventional breeding, genetic transformation is a potential alternative and complementary strategy to accelerate the production of passion fruit cultivars with improved traits.

*Agrobacterium tumefaciens* and particle bombardment are commonly used methods for genetic engineering of plants. However, *Agrobacterium*-mediated transformation is popular in many plant species due to the integration of single-copy transgenes with minimal rearrangements, its high efficiency of transgene integration and simplicity of the equipment used [7, 8]. Genetically modified passion fruit plants by *Agrobacterium*-mediated transformation were first reported about 25 years ago [9]. Since then, limited progress has been achieved in the development of genetic transformation technologies and subsequent gene-function assessments in only three passion fruit germplasm. Trevisan et al. [10] reported transformation of two Brazilian yellow passion fruit (*Passiflora edulis* f*. flavicarpa*) cultivars IAC-275 and IAC-277 using a gene construct for resistance to *Cowpea aphid borne mosaic virus* (CABMV), which resulted in transformation efficiencies of 0.11 and 0.21%, respectively. Transformation of *Passiflora alata* for resistance to CABMV has also been reported with 0.89% transformation efficiency [11]. Recently, Tuhaise et al. [12] documented a transformation efficiency of 0.456% following genetic transformation of Uganda’s yellow passion fruit. The transformation efficiencies in these studies were determined by dividing the number of PCR positive plant lines by the number of explants inoculated, expressed as a percentage. These previous reports involved complicated procedures which took more than 5 months to generate transgenic seedlings. In addition, previous reports on transformation are based on model yellow passion fruit cultivars, which are not farmer-preferred due to low yield. To exploit the desirable traits of farmer-preferred passion fruit varieties, it is important to develop simple, rapid and efficient transformation system for these varieties.

The success of an *Agrobacterium*-mediated transformation system is influenced by many variable parameters including the type of explants, duration of pre-culture of explant, *Agrobacterium* strain, bacterial cell density, infection time, co-cultivation duration, concentration of acetosyringone, cultivars and antibiotic selection [13]. Here we present a transformation protocol for the popular farmer-preferred passion fruit hybrid KPF4 [purple passion fruit (*Passiflora edulis* f. *edulis*) × yellow passion fruit (*Passiflora edulis* f. *flavicarpa*)]. KPF4 is a sweet yellow passion fruit developed and released by Kenya Agricultural Research and Livestock Organization (KARLO). This variety is a farmer-preferred passion fruit due to production of large fruits with sweet and high juice content [14]. It is drought tolerant and well adapted to the coastal lowlands of Kenya [14], a region characterized by erratic rainfall. However, KPF4 is susceptible to passion fruit woodiness disease (PWD), which significantly affects passion fruit production [15], hence genetic improvement of passion fruit is required.

The aim of this study was to optimize factors that affect *Agrobacterium*-mediated transformation of KPF4 and establish a simple *Agrobacterium*-mediated protocol for commercial hybrid passion fruit KPF4. We initially established a simple and rapid plant regeneration protocol from leaf disc explants within 8 weeks. Based on this, an *Agrobacterium*-mediated DNA transformation method for KPF4 was developed. The method is simple and fast and could facilitate future genetic improvement of this important fruit crop. The method also provides an opportunity for introduction of agronomic traits such as resistance to viruses and longer storage of the edible fruits.

## Materials and methods

### In vitro seed germination to generate leaf explants

Ripe fruits of passion fruit variety KPF4 (*Passiflora edulis* f. *edulis* × *Passiflora edulis* f. *flavicarpa*) were sourced from Kenya Agricultural and Livestock Research Organization (KALRO), Thika (GPS coordinates: S0100078, E03704810, 4992 m above sea level). Mature seeds were extracted from ripe fruits of variety KPF4, rinsed with tap water and dried in the sun for 72 h. The seeds were surface-sterilized with 70% (v/v) ethanol for 5 min, followed by 2.5% sodium hypochlorite (v/v) for 20 min and then rinsed four times in double sterile distilled water. An incision of approximately 2 mm was carefully made on the lateral sides of each seed. The seeds were then placed on sterile seed germination medium (SGM; Additional file 1: Table S1). The SGM medium was prepared by dissolving Murashige and Skoog (MS) solid medium with vitamins [16], 2% (w/v) sucrose and the pH was adjusted to 5.8 using 0.1 N NaOH, followed by addition of 0.24% (w/v) gelrite. The medium was then autoclaved at 121 °C for 15 min. The jars with surface-sterilized seeds were incubated at 26 ± 2 °C for germination. Leaves from 21-day-old seedlings were excised, aseptically cut into 6 mm^2^ disks and used as explants for regeneration and transformation experiments.

### Optimization of a regeneration system from KPF4 leaf disc explants

The excised disc explants (6 mm^2^) were placed on shoot induction medium (SIM; MS with vitamins supplemented with 3% (w/v) sucrose and 0.24% (w/v) gelrite; pH adjusted to 5.8 using 0.1 N NaOH). The effect of 6-benzyl amino purine (BAP) at different concentrations (0, 1, 2 and 3 mg L^−1^) was evaluated on regeneration of shoots. Tissue cultures were incubated at 28 ± 2 °C, light intensity of approximately 60 μmole photons m^−2^s^−1^ and a 16 h photoperiod for 4 weeks. After incubation, the effect of different concentrations of BAP was evaluated based on the regeneration frequency and the number of shoots per explant. The regeneration efficiency was calculated as percentage of the number of leaf disc explants with induced shoots per total number of explants cultured. Micro-shoots were transferred onto shoot development medium (SDM; MS with vitamins containing 0.1 mg L^−1^ BAP, 3% (w/v) sucrose and 0.24% (w/v) gelrite, pH adjusted to 5.8 using 0.1 N NaOH) for 2 weeks. Well-developed shoots were transferred as stem cuttings with leaves to rooting initiation and development medium (RIM; MS with vitamins augmented with 0.1 mg L^−1^ naphthaleneacetic acid (NAA), 3% (w/v) sucrose and 0.24% (w/v) gelrite, pH adjusted to 5.8 using 0.1 N NaOH). After 2 weeks, the rooted plantlets were removed from the jars, washed with running tap water to remove agar and transferred into small pots containing sterilized forest soil and sand (1:1) for acclimatization. The humidity of the plants in the pots was maintained high by covering the pots with polyethylene bags.

### *Agrobacterium* strain and plasmid vector

The hypervirulent *Agrobacterium tumefaciens* strain LBA4404 (Invitrogen, USA) containing binary vector pCAMBIA 1301 (http://www.cambia.org.au/) was used for optimization of transformation experiments. The pCAMBIA 1301 contains a hygromycin phosphotransferase (*hpt*) gene as a selection marker and a *gus*A gene with a castor bean catalase intron as a reporter gene, both driven by a CaMV35S promoter (Additional file 1: Figure S1).

### Preparation of *Agrobacterium* suspension cultures

Hypervirulent *Agrobacterium* strain LBA 4404 harbouring pCAMBIA 1301 stored at −80 °C was revived by streaking on Luria–bertani (LB) medium (composed of 10 g L^−1^ tryptone, 5 g L^−1^ yeast extract, 10 g L^−1^ sodium chloride, 15 g L^−1^ agar and pH adjusted to 7.2 with 0.1 N NaOH) plates containing kanamycin (50 mg L^−1^) and rifampicin (50 mg L^−1^). Single colonies obtained from the streaked LB agar petri plates incubated at 28 °C were used to initiate 2 ml LB medium starter cultures. After 48 h shaking at 150 rpm at 28 °C, the starter culture was used as an inoculum to start a bacterial suspension of 20 ml LB with the same antibiotics and grown overnight on a shaker at 150 rpm to obtain an optical density (OD_600_) of 1.0. The bacterial culture was then centrifuged at 3500 rpm for 15 min, the supernatant was poured off and the pellet was re-suspended in 20 ml liquid Murashige and Skoog (MS) with vitamins medium (inoculation medium), supplemented with acetosyringone (Sigma Chemical Co.) at a concentration of 200 μM. The bacteria were further cultivated for 1 h at 25 °C with shaking at 100 rpm. The optical density (OD_600_) of the bacterial suspension was then adjusted to 0.5 using MS with vitamins medium and the suspension was subsequently used for transformation.

### Optimization of parameters affecting *Agrobacterium*-mediated transformation

The factors (bacteria cell density, duration of infection, co-cultivation period and acetosyringone concentration) affecting *Agrobacterium*-mediated transformation were evaluated. For the optical density of bacteria, the pellet was dissolved in the inoculation medium to obtain an OD_600_ of 0.1, 0.25, 0.5, and 0.75. Five different durations of infection (10, 20, 30, 40 and 50 min) of the leaf disks were evaluated. The inoculated leaf disks were co-cultivated for different periods of 0, 1, 2, 3, 4 and 5 days. The effect of acetosyringone (Sigma Chemical Co.) at different concentrations (0, 50, 100, 150, 200, 450 µM) was also tested. The influence of these factors (bacteria density, duration of infection, co-cultivation period and concentration of acetosyringone) on transfer of transgene by *Agrobacterium* to the leaf disc explants and transformation efficiency was evaluated by monitoring transient *gus*A expression after co-cultivation on MS with vitamins supplemented with 3% sucrose, 2 mg L^−1^ benzyl amino purine, BAP, and 0.24% (w/v) gelrite, pH adjusted to 5.8 with 0.1 N NaOH. The experiments were performed using thirty (30) explants for each treatment with three replicates. The obtained data were expressed as percentage of explants showing blue coloration after GUS staining.

## Transformation, selection and regeneration of putative transgenic plants

### Inoculation and co-cultivation of leaf discs

After optimization of factors affecting transformation efficiency, the optimum parameters were used for subsequent *Agrobacterium*-mediated transformation of leaf discs to generate transgenic plants. A total of 300 leaf disc explants were used for each experiment and the experiments were done in triplicates. The leaf disc explants (approximately 6 mm^2^) were injured 10 times using sterile needles and immersed in *Agrobacterium* suspension culture (OD_600_ = 0.5) supplemented with 200 μM acetosyringone followed by gentle shaking at 25 rpm for 30 min at room temperature (23 °C). After inoculation, the leaf disc explants were blotted dry on sterile paper towels and cultured on co-cultivation medium (MS supplemented with 3% (w/v) sucrose, 2 mg L^−1^ benzylaminopurine, BAP, and 0.24% (w/v) gelrite, pH adjusted to 5.8 with 0.1 N NaOH) in Petri plates for 3 days under dark condition at 22 ± 1 °C, light intensity of 60 μmole photons m^−2^ s^−1^ and a photoperiod of 16/8 h. The leaf disc explants were washed four times with sterile liquid co-cultivation medium (MS supplemented with 2 mg L^−1^ BAP and 3% (w/v) sucrose, pH adjusted to 5.8 with 0.1 N NaOH) supplemented with 450 mg L^−1^ cefotaxime, blotted dry on sterile paper towels and cultured onto resting medium (RM; MS with vitamins supplemented with 2 mg L^−1^ BAP, 3% (w/v) sucrose, 2.4% (w/v) gelrite, pH adjusted to 5.8 with 0.1 N NaOH) containing 450 mg L^−1^ cefotaxime for 4 days, 28 °C with 16/8 h photoperiod.

### Antibiotic selection and regeneration of putatively transformed plant lines

After 4 days of resting stage, the transformed explants were transferred onto fresh shoot induction medium (SIM; MS supplemented with 2 mg L^−1^ BAP, 3% (w/v) sucrose and 0.24% (w/v) gelrite, pH adjusted to 5.8 with 0.1 N NaOH) containing 450 and 7.5 mg L^−1^ of cefotaxime and hygromycin, respectively. The explants were incubated for 2 weeks at 28 °C and a 16/8 h photoperiod. The cultures were transferred to fresh SIM supplemented with 450 mg L^−1^ cefotaxime and 7.5 mg L^−1^ hygromycin and incubated further for 2 weeks at 28 °C, light intensity of 60 μmole photons m^−2^s^−1^ and a 16/8 h photoperiod. The developed micro-shoots were placed on shoot development medium (SDM; MS with vitamins supplemented with 0.1 mg L^−1^ BAP, 3% (w/v) sucrose and 0.24% (w/v) gelrite, pH adjusted to 5.8 with 0.1 N NaOH) containing 450 and 7.5 mg L^−1^ of cefotaxime and hygromycin, respectively, for 2 weeks. The stems of putatively transformed shoots were cut off and transferred onto root initiation medium (RIM; MS supplemented 0.1 mg L^−1^ NAA, 3% (w/v) sucrose, 0.24% (w/v) gelrite, pH adjusted to 5.8 with 0.1 N NaOH). The RIM was also supplemented with 7.5 mg L^−1^ hygromycin to confirm the status of the transgenic plants. The leaves of regenerated transgenic plants were used for histochemical β-glucuronidase assays and polymerase chain reaction (PCR) analysis. The transformation efficiency (%) was determined by dividing the number of PCR positive plant lines by the number of leaf disc explants inoculated and expressed as a percentage.

## Analysis of putative transgenic plant lines

### Histochemical β-glucuronidase (GUS) assays

The co-cultivated explants and leaves of putatively transformed plants were utilized for β-glucuronidase histochemical assays [17]. Transient and stable *gus*A gene expression was driven by the cauliflower mosaic virus 35S promoter. Assays were done using transformed explants after 3 days of co-cultivation and on leaves of shoots regenerated on selection medium, respectively. Leaves of regenerated non-transformed shoot plants were used as controls. Briefly, the leaf discs were immersed in GUS staining solution [100 mM sodium phosphate buffer (pH 7.0) containing 2 mM 5-bromo-4-chloro-3-indolyl glucuronide (Duchefa Biochemie, Haarlem, The Netherlands), 0.05 mM potassium ferricyanide, 0.05 mM potassium ferrocyanide, and 0.1% (w/v) Triton X-100], and incubated in the dark at 37 °C overnight. The staining mixture was poured off, 70% (v/v) ethanol was added to remove chlorophyll and the stained plant material was photographed.

### Polymerase chain reaction-based confirmation of putative transgenic lines

Two hundred (200) milligram leaves of in vitro regenerated shoots were used to isolate total genomic DNA using a cetyl trimethyl ammonium bromide (CTAB) procedure [18]. For PCR analysis, the *gus*A gene was amplified using *gus*A-specific primers to confirm the presence of the transgene in regenerated plants. The primers were designed to amplify a 542 bp product and the sequences were: forward primer 5′-TTTAACTATGCCGGGATCCATCGC-3′, and reverse primer 5′-CCAGTCGAGCATCTCTTCAGCGTA-3′.

Polymerase chain reaction (PCR) was carried out in a total amplification reaction contents of 25 µl, which contained 12.5 μl of premix (One*Taq*^®^ Quick-Load^®^ 2 × Master Mix), 9.5 μl DNA- and RNA-free water, 1 µl of 10 µM of both forward and reverse primers and 1 μl of genomic DNA. The PCR amplification was carried out using the following thermocycling conditions: initial denaturation at 94 °C for 10 min followed by 35 cycles of 15 s denaturation at 94 °C, 40 s annealing at 62 °C and 50 s extension at 72 °C, followed by a final extension for 7 min at 72 °C. The PCR products were separated on a 0.8% (w/v) agarose gel (Duchefa Biochemie, Netherlands) containing 1 × Tris–Acetate EDTA buffer. Staining of the amplified products was carried out using 0.5 µg/ml ethidium bromide. The PCR products (4 μl) and loading dye (6 ×) were loaded into each well. The electrophoresis was run at 70 V for 45 min in a Pharmacia biotech horizontal tank. Gels visualization was carried out under a UV transilluminator and gels were photographed by a gel documentation System (Bio Rad).

### Acclimatization of transgenic plants in the glasshouse

Five replicates of regenerated plants approximately 5 cm in length with more than 5 well developed roots were removed carefully from RIM and washed gently with sterilized distilled water to remove any adhered gelrite. The plantlets were transferred to plastic pots (10 cm diameter) containing sterile coconut peat (Grekkon Ltd, Kenya). All pots were covered with clear transparent polyethylene bags to allow in light to be received by the plants and to maintain humidity. The potted plantlets were placed under glasshouse conditions at 28 °C and 70% humidity. The polythene bags were opened gradually. After 2 weeks the polyethylene bags were removed completely and plantlets were watered at regular intervals. Subsequently the plantlets were transferred to larger plastic pots (30 cm × 40 cm) containing sterilized garden soil and sand (1:1) and kept under glasshouse conditions.

### Statistical data analysis

All experiments were performed in three replicates and the experiments were repeated three times. Completely randomized designs (CRD) were used in all experiments. For optimization of factors affecting transformation, the percentage of GUS positive explants was calculated under different conditions of transformation. The statistical data analysis was done using GenStat^®^ Statistical software 15th edition at P = 0.05 using analysis of variance (ANOVA) and Tukey’s test.

## Results

### Establishment of a simple and rapid regeneration method

**Fig. 1:**
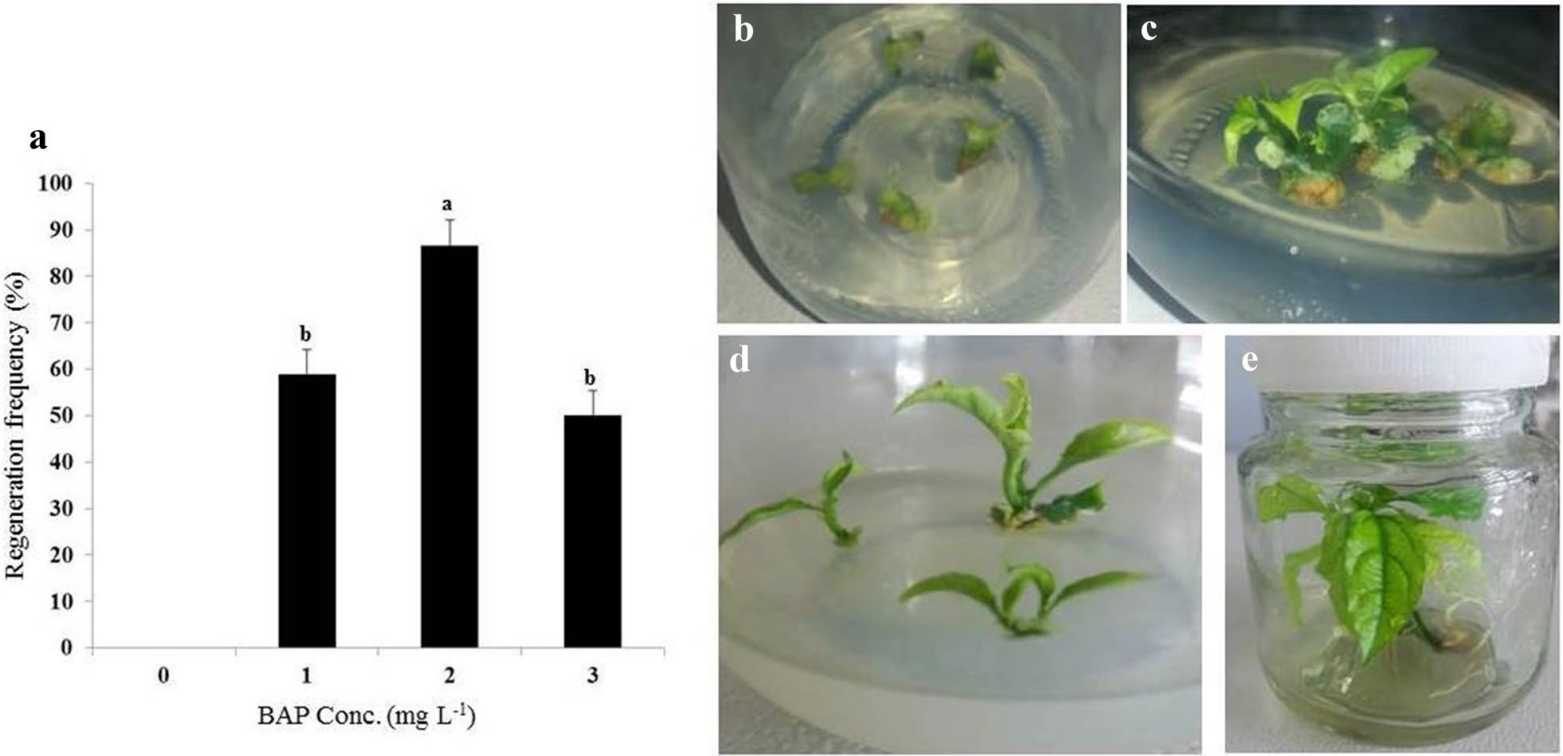
Effects of different concentrations of BAP on induction of shoots from leaf disc explants of passion fruit. **a** The regeneration efficiency on MS with vitamins supplemented with different BAP concentrations. The highest efficiency was achieved with 2 mg L^−1^ BAP and data are mean ± SE. Means having the same letters above bars are not significantly different at P > 0.05 using Tukey’s Honest significant difference test. **b** Shoot bud induction on leaf disc explants, **c** Shoot buds proliferating into shoots, **d** Elongation of induced shoots, and **e** The rooting of regenerated shoots

The objective of this was to establish a simple and fast regeneration procedure to facilitate *Agrobacterium*-mediated cell transformation of hybrid passion fruit KPF4. To establish shoot organogenesis, different BAP concentrations (0, 1.0, 2.0 and 3.0 mg L^−1^) were tested to obtain the optimum concentration. Shoot buds were induced in all the tested concentrations of BAP, though at different regeneration frequencies (Fig. 1a). However, on medium without BAP, there were no shoots induced from the cultured leaf disc explants. The first morphogenetic responses on the cut or injured surfaces were visible after 16–21 days, when shoot buds were observed from the leaf explant (Fig. 1b). The regeneration frequency of 0–86.67% was obtained depending on the concentration of BAP used. A concentration of 2.0 mg L^−1^ BAP resulted in the highest induction of shoots at 86.67 ± 5.53%, which was significantly (p < 0.05) different from the other tested concentrations of BAP (Fig. 1a). Leaf disc explants cultured on MS medium containing 3 mgL^−1^ BAP regenerated the lowest shoots per explant (4.5 ± 0.8 shoots per leaf disc explant) (Fig. 1a).

The micro-shoots (Fig. 1c) regenerated at different concentrations of BAP, did not develop further or elongate after prolonged culturing on the same media for more than 4 weeks. Elongation of micro-shoots was thereafter achieved at 0.1 mg L^−1^ BAP (Fig. 1d). Roots appeared on elongated shoots after 7 days on MS with vitamins augmented with 0.1 mg L^−1^ naphthaleneacetic acid (NAA), 3% (w/v) sucrose and 0.24% (w/v) gelrite. All plantlets had well developed roots (Fig. 1e) after 2 weeks of culture on root initiation and development medium. Fully developed rooted plantlets were transferred to the glasshouse for acclimatization. Plants regenerated through leaf discs were normal and no changes in morphology were observed in the potted plants.

### Optimization of factors influencing *Agrobacterium*-mediated transformation

**Fig. 2:**
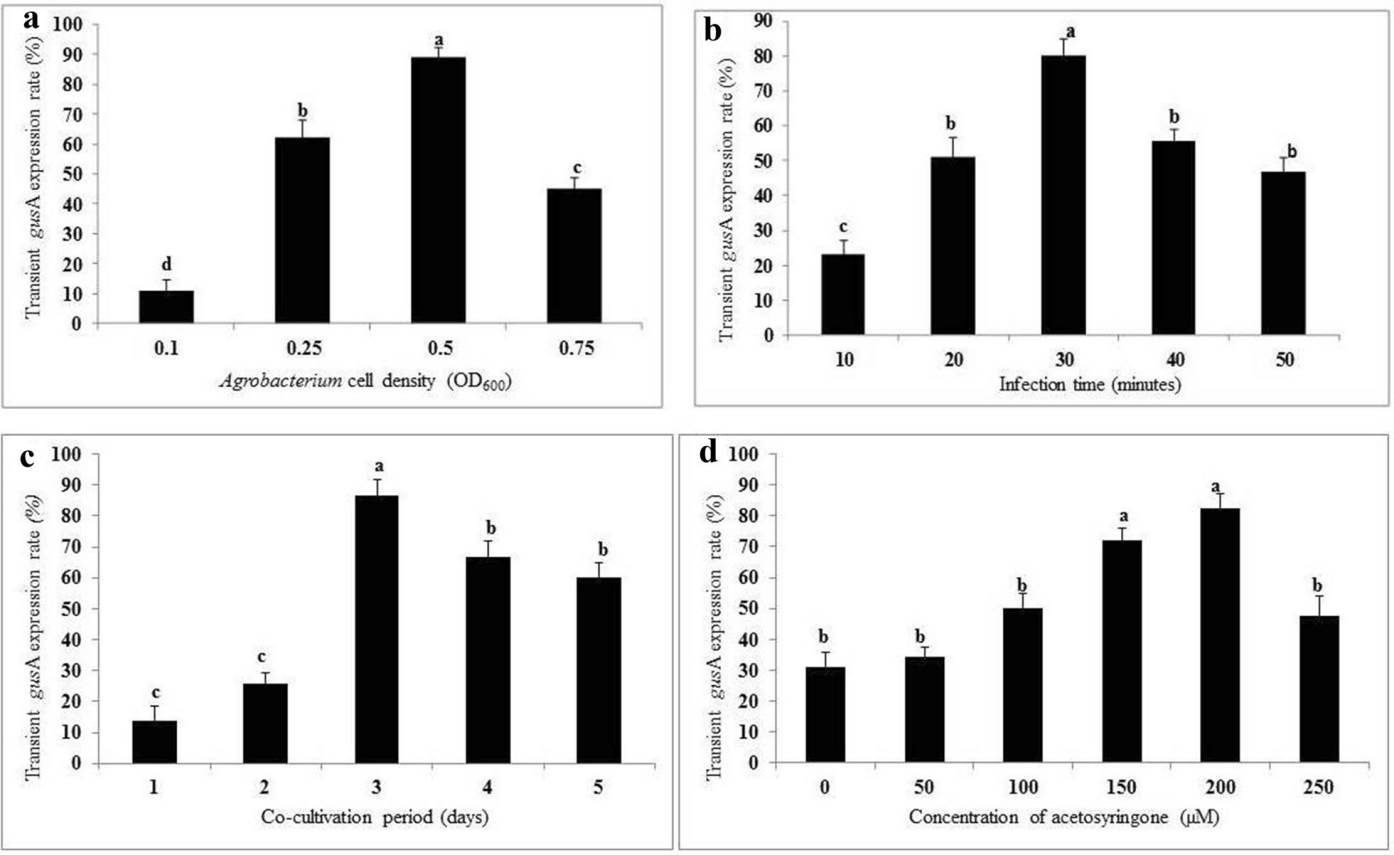
Factors affecting transient *gus*A expression of leaf disc explants of passion fruit using *Agrobacterium* strain LBA4404. **a** Effect of *Agrobacterium* cell density (OD600 values of 0.1, 0.25, 0.5 and 0.75); **b** Effect of infection time (10, 20, 30, 40 and 50 min); **c** Effect of co-cultivation period (1, 2, 3, 4 and 5 days); **d** Effect of concentration of acetosyringone (0, 50, 100, 150, 200 and 450 µM). Each experiment consisted of 30 leaf disc explants and experiments were done in triplicates. Treatments (mean ± SE) having the same letters are not significantly different at P > 0.05 using Tukey’s significant difference test

In this study, the effect of *Agrobacterium* cell density (LBA4404 harbouring pCAMBIA 1301), time of infection, period of co-cultivation and concentration of acetosyringone on transformation efficiency of KPF4 was evaluated based on expression of an intron-containing *gus*A. For *Agrobacterium* density, there was a significant increase in *gus*A expression with increasing *Agrobacterium* cell density with an OD_600_ from 0.1 to 0.5. The highest *gus*A expression of the inoculated leaf disc explants (88.9%) was obtained with LBA4404 at OD_600_ of 0.5. Therefore, bacteria cell density of OD_600_ = 0.5 was selected for *Agrobacterium*-mediated transformation of KPF4. The increase in bacterium cell density at OD_600_ beyond 0.5, led to a significant reduction in the percentage of leaf explants (44.9%) displaying *gus*A expression. The best optical cell density for delivery of the transgene into plant tissues was 0.5 at OD_600_ (Fig. 2a).

The duration of *Agrobacterium* infection of leaf explants had a significant influence on *gus*A expression. At the optimum cell density (OD_600_ = 0.5), the highest percentage of explants (80%) displaying *gus*A expression was obtained at 30 min *Agrobacterium* infection time. Either decrease or increase of *Agrobacterium* infection time beyond the optimum (30 min), significantly reduced the percentage of explants showing *gus*A expression (Fig. 2b).

The duration of co-cultivation had a significant effect on transient *gus*A expression in inoculated leaf disc explants of KPF4. There was a significant increase in transient *gus*A expression with increasing number of days of co-cultivation from 1 to 3. The optimum transient *gus*A expression (86.7%) was observed in leaf explants co-cultivated for 3 days post-infection. *Gus*A expression of leaf explants co-cultivated for 3 days was significantly different (p≤ 0.05) from the other co-cultivation periods. The increase in co-cultivation period beyond 3 days significantly decreased the rate of transient *gus*A expression (Fig. 2c) and necrosis/browning of the leaf explants was observed.

The use of acetosyringone for induction of virulence genes in *Agrobacterium* was found to significantly influence transient *gus*A expression of inoculated leaf disc explants of KPF4. The concentration of acetosyringone had a significant (p ≤ 0.05) effect on transient *gus*A expression. The optimum transient *gus*A expression of leaf explants (82.2%) was obtained with acetosyringone at concentration of 200 µM. However, transient *gus*A expression at 200 µM acetosyringone was not significantly (p > 0.05) different from 150 µM. Acetosyringone concentration more than 200 µM in the co-cultivation medium significantly reduced the percentage of explants (47.7%) displaying transient *gus*A expression (Fig. 2d).

### Genetic transformation and regeneration of putative transgenic plant lines

**Fig. 3:**
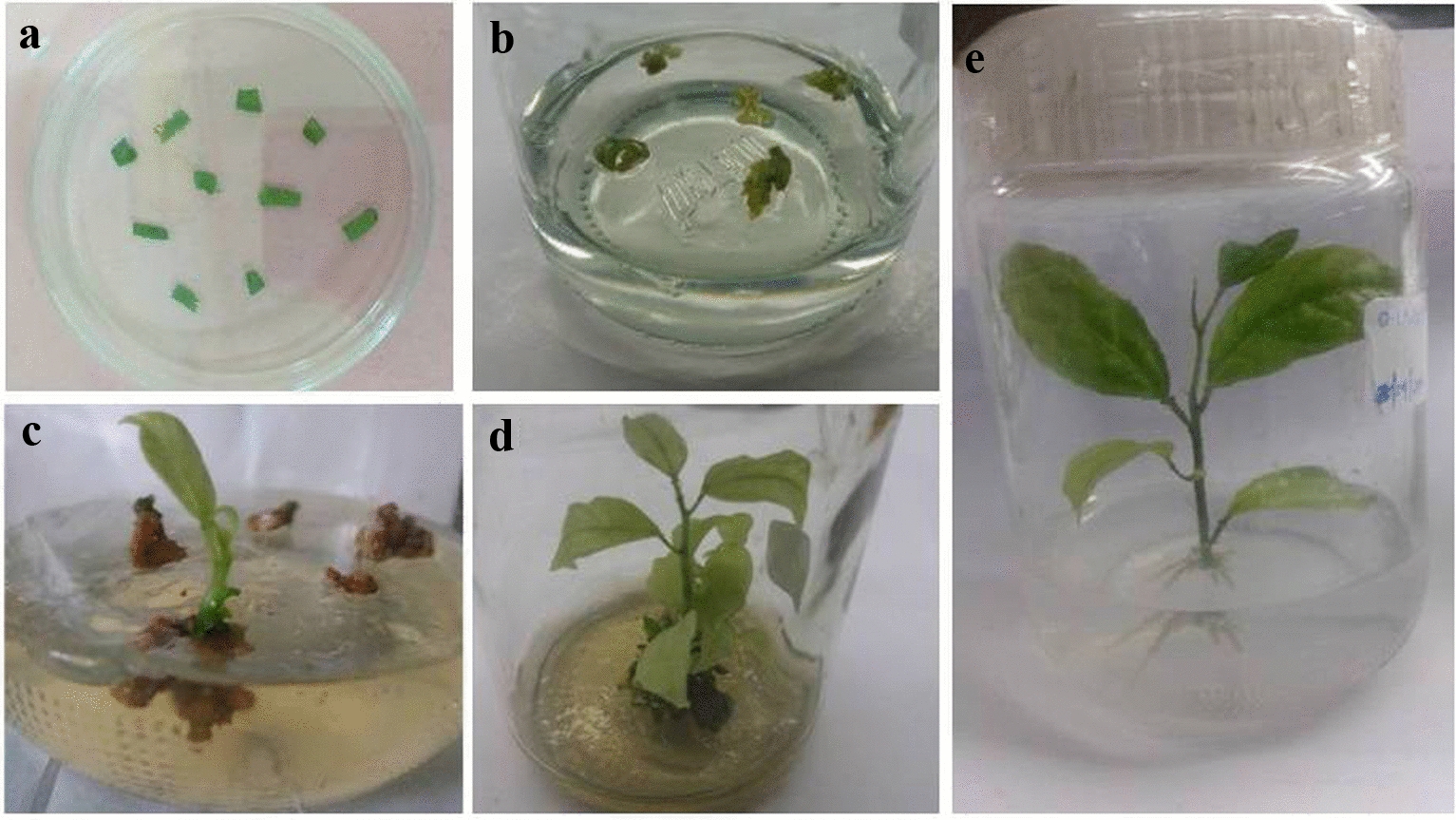
*Agrobacterium*-mediated transformation of leaf disc explants of passion fruit KPF4 using *Agrobacterium* strain LBA4404 harboring pCAMBIA1301. **a** Culture of inoculated leaf disc explants on resting medium; **b** One-week-old inoculated explants on shoot bud induction medium with selection agent (7.5 mg L^−1^ hygromycin); **c** Micro-shoots of leaf explants on SIM containing selection agent (7.5 mg L^−1^ hygromycin) for 3 weeks; **d** Elongated shoots after 4 weeks of culture on SDM containing selection agent (7.5 mg L^−1^ hygromycin); **e** Rooting test on RIM supplemented with 7.5 mg L^−1^ hygromycin for 2 weeks

Successful *Agrobacterium*-mediated transformation of leaf disc explants of passion fruit hybrid KPF4 was developed based on the optimized conditions of transient *gus*A gene expression. Following transformation (*Agrobacterium* optical cell density OD_600_ of 0.5, 30 min of *Agrobacterium* infection time, 200 µM of acetosyringome and 3 days of co-cultivation), the explants were washed, placed on recovery phase (Fig. 3a) for 4 days followed by transfer to shoot bud inducing medium containing selection agent (7.5 mg L^−1^ hygromycin). The non-transformed leaf explants turned brown within 7 days and the transformed leaf explants had green segments (Fig. 3b) which formed shoot buds after 3 weeks of culture. After 4 weeks, induced shoot buds developed into micro-shoots (Fig. 3c). The non-transformed leaf disc explants turned necrotic and finally died when placed on selective medium (Fig. 3c). The transfer of micro-shoots onto MS augmented with reduced BAP concentration (0.1 mg L^−1^ BAP) and selection agent (7.5 mg L^−1^ hygromycin) developed into fully elongated shoots (Fig. 3d) after 2 weeks of culture. Roots were initiated after 7 days on elongated shoots cultured on root initiation and development medium and fully developed roots were formed after 2 weeks of culture (Fig. 3e). All the PCR-positive shoots formed roots in root induction medium. Following the three transformation experiments, a total of 900 leaf disc segments of *P. edulis* were infected with *Agrobacterium*, 79 explants survived on selection medium, 18 shoot buds were induced on shoot induction medium (SIM), 10 hygromycin resistant elongated shoots were obtained on shoot development medium (SDM), and 6 positive transgenic shoots developed roots on root-inducing medium (RIM). The transformation efficiency obtained in this study was 0.67% (Table 1).

**Table 1:** Generation of transgenic plants of hybrid passion fruit KPF4

Transformation experiment	No. of explants infected with *Agrobacterium* harboring pCAMBIA1301	No. of explants surviving on selection medium	No. of explants forming shoot buds on SIM with selection agent	No. of shoots developed on SDM with selection agent	Rooting on RIM with selection agent	No. of transgenic lines with GUS expression	No of PCR-positive plant lines	Transformation efficiency (%)
1	300	18	5	3	2 (66.67%)	2	2	0.67
2	300	29	6	4	1 (25%)	1	1	0.33
3	300	37	7	3	3 (100%)	3	3	1

**Fig. 4:**
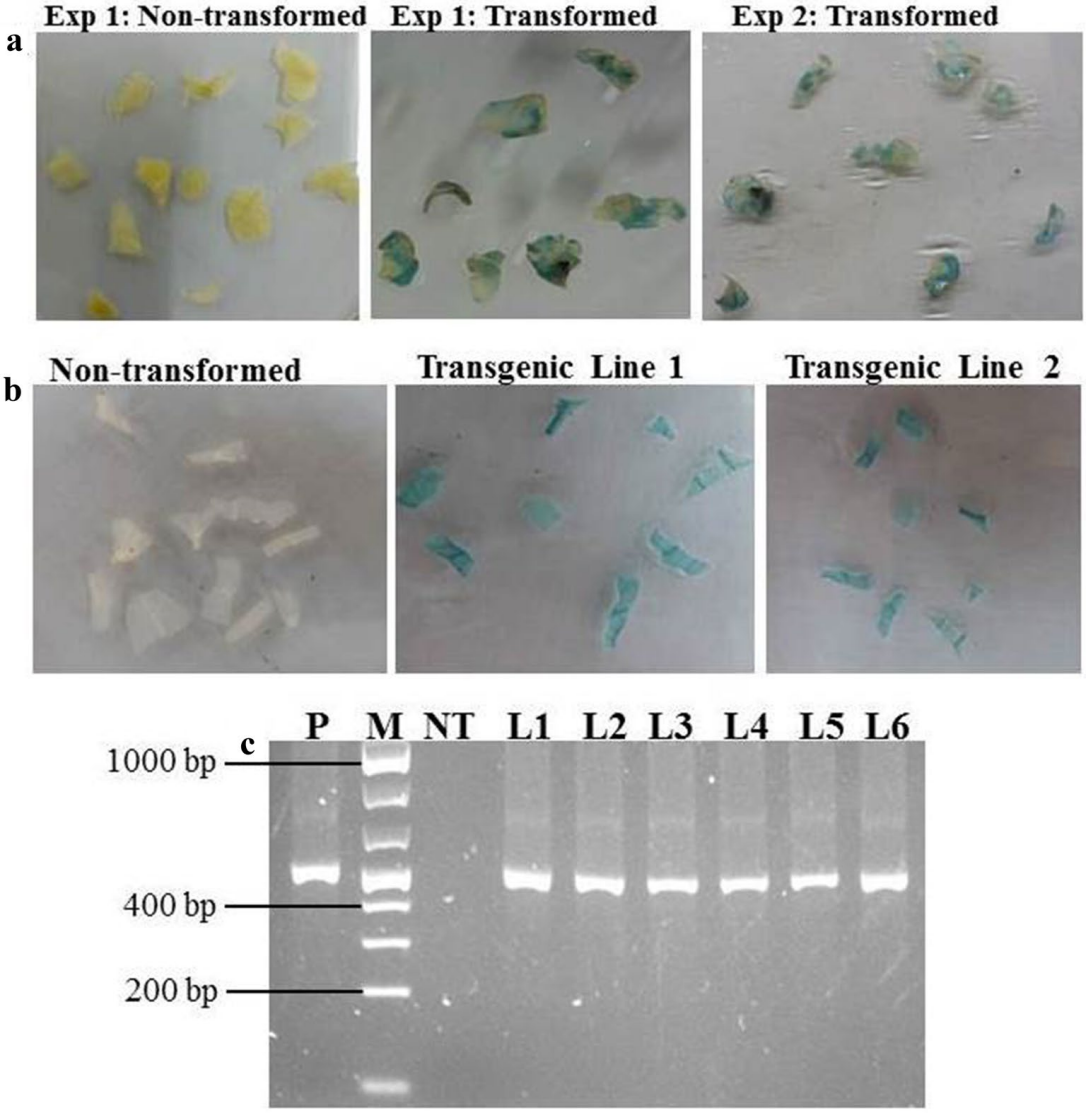
Histochemical β-glucuronidase staining and PCR analysis of transformed leaf discs and transgenic plants. **a** Histochemical GUS staining of leaf discs after co-cultivation for 3 days; **b** Stable histochemical GUS staining of leaf segments of different transgenic plant lines and non-transformed control plant; and **c** PCR amplification of *gus*A gene fragment of DNA isolated from 6 transgenic plant lines. *P* = plasmid DNA, M = 1 kb molecular weight marker (Fisher Thermo scientific); NT = Non-transformed control plant; and L1–L6 = Transgenic plant lines 1 to 6, respectively

GUS expression was used to check the progress of transformation and blue coloration was detected in KPF4 leaf disc explants as early as 4 days post-infection with LBA4404 harbouring pCAMBIA1301. Transformed leaf explants were verified by histochemical GUS assay and 100% of explants showed transient *gus*A gene expression (Fig. 4a). A uniform blue staining (Fig. 4b) was observed in all the leaf discs of all the regenerated putatively transgenic plants. No blue staining (Fig. 4b) was obtained in leaves of non-transformed control plants regenerated without selection.

PCR analysis with primers designed to amplify a fragment of the *gus*A reporter gene was performed on DNA isolated from control plants regenerated from leaf disc explants that did not undergo infection and putative transgenic plant lines regenerated from explants infected with LBA4404 harboring pCAMBIA1301. Expected band sizes of 542 bp equivalent to a fragment of the *gus*A reporter gene were amplified from genomic DNA of all putatively transformed plant lines. No amplicon was obtained in non-transgenic control plants (Fig. 4c). The PCR results showed that all the regenerated six passion fruit lines were transgenic plants.

**Fig. 5:**
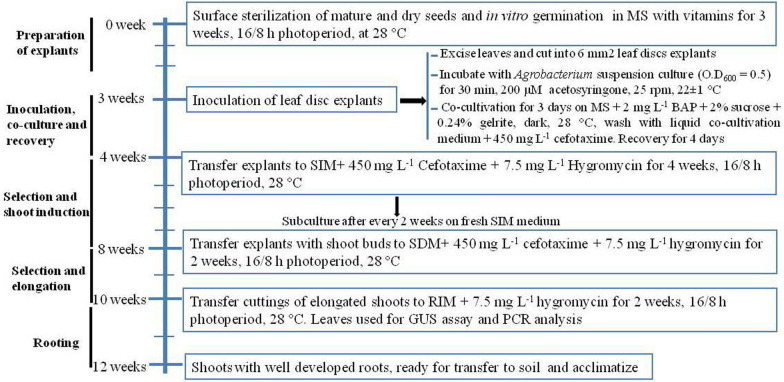
A flow diagram for *Agrobacterium*-mediated transformation method of passion fruit hybrid KPF4 using leaf disc explants

Based on the findings obtained in the current study, we present a simple and fast protocol for *Agrobacterium*-mediated transformation of *Passiflora edulis* Sims using leaf disc explants. After *Agrobacterium* infection, it takes approximately 8 weeks for transgenic lines to be ready for transfer to the soil. A summary of the established protocol is shown in Fig. 5.

## Discussion

A fundamental first step for the successful establishment of a genetic transformation system in plants is an efficient plant regeneration procedure. In this study, we developed a simple and quick regeneration system of hybrid passion fruit KPF4. Plantlets formed on the leaf disc segments within 4 weeks. BAP concentrations tested had the potential to successfully induce shoots, nevertheless, the induction results indicated that 2.0 mg L^−1^ BAP was the optimum concentration with a higher efficiency on shoots induction compared to the other concentrations. BAP has been reported as an efficient cytokinin that induces regeneration of shoots in many plants including passion fruit species such as *P. edulis f. flavicarpa*, *P*. *caerulea*, *P. alata* and *P. setacea* [19–21]. However, on medium containing 2.0 mg L^−1^ BAP, the induced shoots of KPF4 did not proliferate or elongate and the problem became severe after six weeks of culture. To optimize the protocol, the leaf explants were cultured on medium containing 2.0 mg L^−1^ BAP to induce shoot buds for 4 weeks and then transferred to MS medium with reduced BAP (0.1 mg L^−1^) for elongation of shoots.

*Agrobacterium* cell density is a fundamental factor influencing genetic transformation system [22]. Different *Agrobacterium* cell densities used to inoculate the leaf disc explants of KPF4 had an effect on the level of transient GUS expression in hybrid passion fruit KPF4. The optimum transient GUS expression was achieved with bacterium OD_600_ of 0.5. At higher concentration of *Agrobacterium*, browning/necrosis was observed in leaf disc explants and finally death. This confirms previous reports that higher densities of *A. tumefaciens* results in tissue damage and hence reduction in the transformation efficiency [23, 24]. Higher bacterium cell density can cause uncontrolled growth of *Agrobacterium* thus limiting the survival of the explants and subsequent reduction in transformation efficiency. Alfenas et al. [25] used optical density of 0.4 to successively transform yellow passion fruit. In studies of other plant species such as rice, wheat, maize, soybean, tea and jute, the optical densities of *Agrobacterium* suspension cultures ranged from 0.1 to 1.0 depending on the genotype, *Agrobacterium* strain and plant species [26–32].

The transfer of T-DNA from *Agrobacterium* to the plant genome during *Agrobacterium*-mediated genetic transformation process is time-dependent and therefore both time of infection and co-cultivation duration affect the transformation efficiency [30]. Varying the inoculation durations had an influence on the transfer of T-DNA from *Agrobacterium* to plant cells and 30 min infection time at an OD_600_ of 0.5 was optimum for KPF4 leaf disc explants, similar to reports by Manders et al. [9] in yellow passion fruit, Jha et al. [33] and Zhao et al. [34] in *Pennisetum glaucum* and rice, respectively. In a previous study, Trevisan et al. [10] reported 20 min as the most suitable infection time for successful transformation of yellow passion fruit. This difference in infection time would be due to the different *Agrobacterium* strains used. Therefore, it is important to optimize the infection times for different *Agrobacterium* strains for each plant species and cultivar and it is better to select a low concentration with high infection ability. Diverse infection times in other plant species such as sorghum, maize and wheat has been reported ranging from 5 min (for sorghum, maize) to 50 min (for wheat) indicating that infection time varies with the plant species under investigation.

Co-cultivation period is important in *Agrobacterium*-mediated transformation because during this step the T-DNA is transferred into the genome of host plants [35, 36]. The co-cultivation period significantly influenced the rate of transient *gus*A expression in KPF4 leaf disc explants. Three days of co-cultivation resulted in the highest transient *gus*A expression rate, similar to previous reports in passion fruit [9, 10, 12]. Shorter co-cultivation periods (1 to 2 days) resulted in lower rates of transient *gus*A expression probably due to inadequate time for maximum transfer of T-DNA from *Agrobacterium* into the plant genome [36]. Co-cultivation for more than 3 days resulted to bacterial overgrowth and browning/necrosis of the leaf disc explants. Co-cultivation periods ranging from 2 to 5 days have been reported to be suitable in several plant transformation studies such as okra [30], yam [37], rice [38], wheat [30], maize [39] and soybean [31]. However, a longer co-cultivation period of 15 days has been documented in a study on sunflower (*Helianthus annuus* L.).

Acetosyringone, a phenolic product produced from plant wounds and has been frequently used in *Agrobacterium*-mediated transformation of plant species during infection and co-cultivation to improve transformation efficiency [40–43]. Acetosyringone induces the activation and expression of virulence genes in *Agrobacterium* required for plant genetic transformation [43]. Depending on the plant species and *Agrobacterium* strain, the concentration of acetosyringone used for transformation varied from 20 to 200 µM [27, 41, 42, 44]. In the present study, addition of acetosyringone into the *Agrobacterium* infection medium significantly increased the rate of transient *gus*A expression of inoculated leaf disc explants. Exogenous addition of 200 µM acetosyringone resulted in the highest transient *gus*A expression and this is in agreement with previous findings in passion fruit [10] and other plant species including sweet potato [45], orchid [46] and banana [47].

The optimization of factors affecting *Agrobacterium*-mediated transformation based on the rate of transient *gus*A expression allowed the establishment of a protocol for stable transformation of commercial hybrid passion fruit KPF4. The genetic transformation efficiency in present study was 0.67%. Other studies reported that transformation efficiencies of the two Brazilian passion fruit varieties IAC-275 and IAC-277 were 0.11% and 0.21%, respectively [10]. Monteiro-Hara et al. [48], documented transformation efficiencies of 0.67% and 0.19% for the same Brazilian varieties reported by Trevisan et al. [10]. A higher transformation efficiency (0.89%) was obtained by Correa et al. [11] on *Passiflora alata*. Recently, Tuhaise et al. [12] reported a transformation efficiency of 0.46% for Ugandan yellow varieties of passion fruit. All these previous reports involved complicated procedures which require more than 5 months to regenerate transgenic plants. The protocol established in this study for passion fruit hybrid KPF4 is simple and quick as it takes 3 months to regenerate transgenic plants.

In the current study, 6 out of the 10 shoots were positive in the rooting test. The non-transgenic shoots did not root in RIM supplemented with 7.5 mg L^−1^ hygromycin. This confirms the reliability of rooting procedure as a rapid second stage selection of transgenic plants as reported in studies of other plant species [49]. Confirming the transgenic plants by use of GUS assays and PCR analysis are other effective ways of eliminating false positive transformants. All the rooted plants in this study showed *gus*A expression as determined by GUS staining and the presence of the transgene was confirmed by PCR. This indicates that the intron-containing *gus*A gene was successfully inserted into the genome of the 6 obtained KPF4 lines. To the best of our knowledge, this is the first report of the successful transformation of commercial passion fruit hybrid KPF4. The establishment of transformation protocol for KPF4 will be critical in initiating its genetic improvement through transgenic approaches.

## Conclusions

The present study established a simple and rapid *Agrobacterium*-mediated transformation protocol for passion fruit hybrid KPF4. Stable transgenic plants which showed presence and expression of *gusA* were regenerated within 12 weeks from leaf disc explants. The protocol can be used to insert beneficial traits into passion fruit hybrid KPF4 to create transgenic plants. This method will also be useful for stacking traits such as virus resistance and longer storage of edible fruits.

## Supplementary information

**Additional file 1: Table S1.** Composition of various media used for in vitro regeneration and *Agrobacterium*-mediated transformation of KPF4 leaf explants. **Figure S1.** Representation of T-DNA region of the binary vector pCAMBIA1301 used for genetic transformation of *Passiflora edulis* Sims. *NOS PolyA* Nopaline synthase polyadenylation signal (terminator), *gus*A β-glucuronidase  reporter gene containing an intron, *35SP* CaMV35S promoter, *MCS* multiple cloning site, *hpt* hygromycin phosphotransferase gene, and *35S PolyA* polyA cauliflower mosaic virus 35S terminator. Both the selection marker and reporter gene are under the control of the CAMV35S promoter.

## Data Availability

The datasets used and analyzed in this study are available from corresponding author on reasonable request. KPF4 variety seeds are available from Kenya Agricultural Research and Livestock Organization (KARLO). LBA4404 and pCAMBIA 1301 are available from Department of Biochemistry, University of Nairobi.
