# Supplementary material for: A simple and fast Agrobacterium-mediated transformation system for passion fruit KPF4 (Passiflora edulis f. edulis × Passiflora edulis f. flavicarpa)
Source: Plant Methods. 2020 Oct 16;16:141. doi: 10.1186/s13007-020-00684-4 (PMC7565748; doi:10.1186/s13007-020-00684-4)
Supplement: Supplementary file 1 — Additional file 1: Table S1. Composition of various media used for in vitro regeneration and Agrobacterium-mediated transformation of KPF4 leaf explants. Figure S1. Representation of T-DNA region of the binary vector pCAMBIA1301 used for genetic transformation of Passiflora edulis Sims. NOS PolyA Nopaline synthase polyadenylation signal (terminator), gusA β-glucuronidase reporter gene containing an intron, 35SP CaMV35S promoter, MCS multiple cloning site, hpt hygromycin phosphotransferase gene, and 35S PolyA polyA cauliflower mosaic virus 35S terminator. Both the selection marker and reporter gene are under the control of the CAMV35S promoter. [file 13007_2020_684_MOESM1_ESM.doc]

**Additional file 1**

**A simple and fast *Agrobacterium*-mediated transformation system for passion fruit KPF4 (*Passiflora edulis* f. *edulis* × *Passiflora edulis* f. *flavicarpa*)**

**Authors and affiliations**

**Lydia K. Asande^1, 2^, Richard O. Omwoyo^1^, Richard O. Oduor^3^, Evans N. Nyaboga^2^**

^1^Department of Plant Science, Kenyatta University, P.O. Box 43844 – 00100, Nairobi, Kenya ^2^Department of Biochemistry, University of Nairobi, P.O Box 30197 – 00100, Nairobi, Kenya

^3^Department of Biochemistry and Biotechnology, Kenyatta University, P. O. Box 43844 – 00100 Nairobi, Kenya

**Additional file 1: Table S1** Composition of various media used for *in vitro* regeneration and *Agrobacterium-*mediated transformation of KPF4 leaf explants

| **Media name** | **Composition** |
| --- | --- |
| Seed germination medium (SGM) | 1 × MS^a^ salts with vitamins, 2% (w/v) Sucrose, 0.24% (w/v) Gelrite, pH 5.8 |
| Shoot induction medium (SIM) | 1 × MS salts with vitamins, 3% (w/v) Sucrose, 0.24% (w/v) Gelrite,1.0 - 3.0 mg L^-1^ BAP, pH 5.8 |
| Shoot Development Medium (SDM) | 1 × MS salts with vitamins, 3% (w/v) Sucrose, 0.24% (w/v) Gelrite, 0.1 mg L^-1^  BAP, pH 5.8 |
| Root induction (RIM) | 1 × MS salts with vitamins, 3% (w/v) Sucrose, 0.24% (w/v) Gelrite, 0.1 mg L^-1^  NAA, pH 5.8 |
| Co-cultivation medium (CM) | 1 × MS salts with vitamins, 3% (w/v) Sucrose, 0.24% (w/v) Gelrite, 2 mg L^-1^ BAP, pH 5.8 |
| Resting medium (RM) | 1 × MS salts with vitamins, 3% (w/v) Sucrose, 0.24% (w/v) Gelrite, 2 mg L^-1^  BAP, 450 mg L^-1^ Cefotaxime, pH 5.8 |
| Luria Bertani medium (LB) | 5 g L^-1^ Yeast extract, 10 g L^-1^ Tryptone, 10 g L^-1^ Sodium chloride, 15 g L^-1^ Agar, pH 7.2 |

^a^Murashige and Skoog [16], BAP 6-benzylaminopurine

MS salts with vitamins, BAP, sucrose, gelrite and Luria Bertani medium were obtained from Duchefa Biochemie, Haarlem, The Netherlands.

**Additional Figure**

**
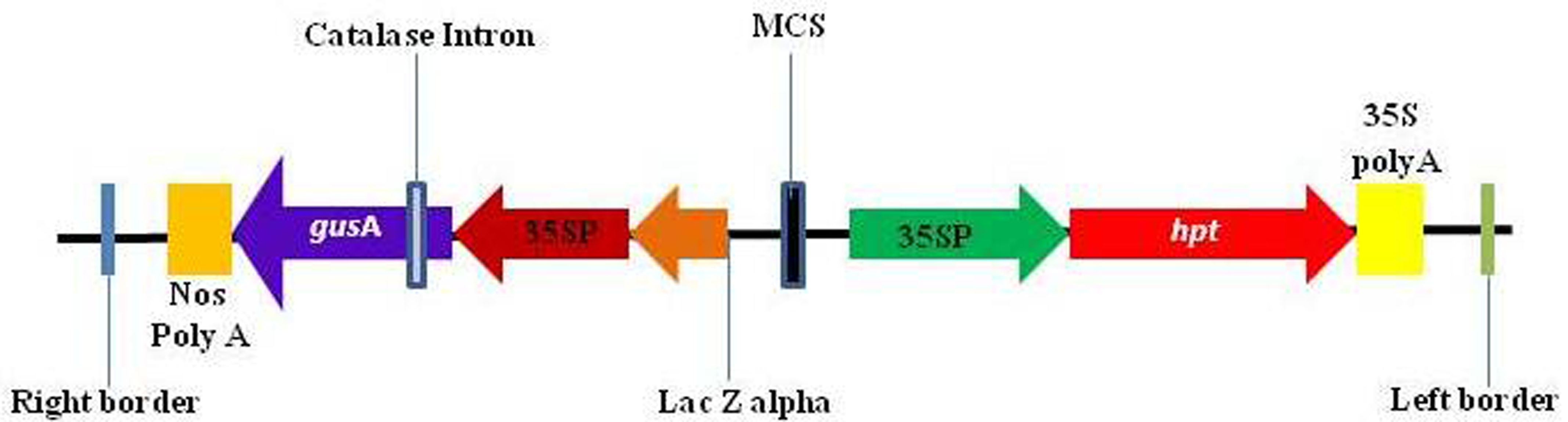
**

**Fig S1** Representation of T-DNA region of the binary vector pCAMBIA1301 used for genetic transformation of *Passiflora edulis* Sims. NOS PolyA - Nopaline synthase polyadenylation signal (terminator), *gus*A - β-glucuronidase  reporter gene containing an intron, 35SP - CaMV35S promoter, MCS - multiple cloning site, *hpt* - hygromycin phosphotransferase gene, and 35S PolyA – polyA cauliflower mosaic virus 35S terminator. Both the selection marker and reporter gene are under the control of the CAMV35S promoter.
